# Moving Beyond LDL-C and Non-HDL-C: Apolipoprotein B as the Stronger Lipid-Related Predictor of Coronary Artery Disease in Statin-Treated Patients

**DOI:** 10.3390/diagnostics15233002

**Published:** 2025-11-26

**Authors:** Raul-Alexandru Jigoranu, Ovidiu Mitu, Alexandru-Dan Costache, Alexandru Oancea, Radu-Stefan Miftode, Ana Maria Buburuz, Amin Bazyani, Paul Simion, Radu Sebastian Gavril, Petru Cianga, Mihail Sebastian Harnau, Viviana Onofrei, Antoniu Octavian Petris, Irina-Iuliana Costache Enache, Florin Mitu

**Affiliations:** 1 Faculty of Medicine, University of Medicine and Pharmacy “Grigore T. Popa”, 700115 Iasi, Romania; alexandru.jigoranu@umfiasi.ro (R.-A.J.); adcostache@yahoo.com (A.-D.C.); oancea.alexandru-florinel@email.umfiasi.ro (A.O.); radu-stefan.miftode@umfiasi.ro (R.-S.M.); ana-maria.buburuz@umfiasi.ro (A.M.B.); aminbazyani@gmail.com (A.B.); rgavril87@yahoo.com (R.S.G.); petru.cianga@umfiasi.ro (P.C.); mihail-sebastian.harnau@umfiasi.ro (M.S.H.); viviana.aursulesei@umfiasi.ro (V.O.); antoniu.petris@umfiasi.ro (A.O.P.); mitu.florin@yahoo.com (F.M.); 2 Department of Cardiology, “St. Spiridon” Emergency County Hospital, 700111 Iasi, Romania; simion_alexis@yahoo.com; 3 Department of Cardiovascular Rehabilitation, Clinical Rehabilitation Hospital, 700661 Iasi, Romania; 4 Laboratory of Immunology, "St. Spiridon" Emergency County Hospital, 700111 Iași, Romania; 5 Romanian Academy of Medical Sciences, 030167 Bucharest, Romania; 6 Romanian Academy of Scientists, 050045 Bucharest, Romania

**Keywords:** apolipoprotein B, chronic coronary syndrome, atherosclerosis, biomarkers

## Abstract

**Background/Objectives:** Coronary artery disease (CAD) remains the leading cause of death primarily in patients over 65 years old, with an increasing incidence, especially in Eastern European countries. Primary and secondary prevention protocols are based on a large number of cardiovascular (CV) risk factors, but low-density lipoprotein cholesterol (LDL-C) remains the main treatment target and one of the central determinants of CV risk. Apolipoprotein B (apoB) is the main structural protein in all atherogenic lipoproteins, and, unlike LDL-C, which only reflects the cholesterol content of LDL, apoB directly quantifies the total number of all circulating atherogenic particles. Over the past decade, a growing amount of data has supported the utility of apoB for CV risk assessment; however, its superiority over LDL-C remains unclear. Our study aimed to investigate the predictive value of apoB for both the presence and the severity of CAD in a statin-treated cohort from an Eastern European hospital and to compare it with standard lipid biomarkers. **Methods:** A total of 121 statin-treated patients, who were evaluated using coronary angiography, were consecutively enrolled and subdivided into three groups: 52 patients with significant coronary artery disease (S-CAD), 36 patients with non-significant coronary artery disease (NS-CAD), and 33 patients without coronary atherosclerosis (N-CAD). Apolipoprotein B was measured at the moment of enrollment using the immunoturbidimetric assay. **Results:** The mean values of LDL-C, TC, non-HDL-C, and apoB increased progressively across the three studied groups. Unlike traditional lipid biomarkers, apoB levels differed significantly not only between N-CAD and S-CAD, but also between N-CAD and NS-CAD. The diagnostic superiority of apoB extended beyond group mean differences, as it also demonstrated the strongest correlation with CAD severity. ApoB showed a moderate correlation with the Gensini score (r = 0.43, *p* < 0.001), which was markedly higher compared to LDL-C, TC, or non-HDL-C, all of which presented only a weak correlation (r = 0.26, r = 0.23, and r = 0.28, respectively). Additionally, in a logistic regression analysis, apoB demonstrated the highest predictive power for the presence of significant CAD (per SD increase: OR 2.386, 95% CI 1.52–3.75, *p* = 0.000), and it was the only biomarker able to predict left main disease (per SD increase: OR 2.433, 95% CI 1.38–4.30, *p* = 0.002) and three vessel disease (per SD increase: OR 1.639, 95% CI 1.012–2.654, *p* = 0.044). Residual apoB was also calculated and remained significantly associated with the presence of coronary atherosclerosis. **Conclusions:** ApoB proved to be a reliable predictor for CAD, independent of LDL-C. Compared to standard lipid biomarkers, apoB was superior in detecting NS-CAD and showed a better correlation with the severity of CAD. Additionally, in our study, only apoB was significantly correlated with left main disease and three vessel disease.

## 1. Introduction

Coronary artery disease (CAD) remains the leading cause of death, especially in patients over 65 years old. However, secondary to a rapidly increasing incidence of CV risk factors among younger individuals, cardiovascular (CV) events tend to become more and more prevalent, especially in Eastern European countries [1]. For this reason, enhancing the effectiveness of existing CV disease screening strategies and primary prevention programs constitutes a public health priority. Over the past decades, a large number of CV risk factors have been identified, and low-density lipoprotein cholesterol (LDL-C) has been one of the central figures in CV disease prevention guidelines worldwide. Other modifiable risk factors, like hypertension, smoking, obesity, or sedentarism, are also highly addressed in the available guidelines; however, during the past years, more and more research was centered on dyslipidemia treatment, LDL-C becoming the main therapeutic target [2].

Despite the unquestionable usefulness of LDL-C for CV prevention, there has been a growing consensus among major CV societies that apolipoprotein B (apoB) is the more accurate single measure of atherogenic lipoprotein particle burden. Unlike LDL-C, which reflects cholesterol content, apoB directly quantifies the number of circulating atherogenic particles and is therefore less influenced by variations in particle cholesterol content—a feature particularly relevant in conditions such as hypertriglyceridemia, metabolic syndrome, diabetes mellitus, and obesity. The 2019 European Society of Cardiology/European Atherosclerosis Society (ESC/EAS) guidelines recommend apoB as an alternative to LDL-C for risk assessment, with treatment goals of <65 mg/dL for very-high-risk and <80 mg/dL for high-risk individuals [2]. The 2021 Canadian Cardiovascular Society (CCS) guidelines allow apoB to replace LDL-C as the primary therapeutic target [3]. Meanwhile, the 2022 ACC Expert Consensus Decision Pathway recognizes apoB ≥ 130 mg/dL as a risk-enhancing factor warranting more intensive therapy [4]. Most recently, the 2024 expert consensus commentary emphasized apoB’s superiority over LDL-C in predicting risk and advocated for its routine integration into clinical practice [5]. Collectively, these recommendations underscore the emerging role of apoB not only as a superior risk marker but also as a central therapeutic target in contemporary lipid management.

Apolipoprotein B is the main structural protein in all atherogenic lipoproteins, including very-low-density lipoprotein (VLDL), intermediate-density lipoprotein (IDL), low-density lipoprotein (LDL), and lipoprotein(a) [Lp(a)] [6]. There are two isoforms: apoB-100, synthesized in the liver, which contains 4536 amino acids and has a molecular weight of approximately 550 kDa; and apoB-48, synthesized in the intestine through mRNA editing, which represents the N-terminal 48% of the apoB-100 sequence [7]. Each atherogenic lipoprotein particle contains exactly one apoB molecule, making its plasma concentration a direct measure of the circulating particle number [8]. Also, apoB-100 serves as the ligand for the LDL receptor, facilitating the hepatic clearance of LDL particles. However, when particle numbers are elevated, the increased endothelial retention of apoB-containing lipoproteins occurs, driven largely by electrostatic interactions between positively charged apoB domains and negatively charged glycosaminoglycans within the arterial intima [9]. This retention initiates lipid accumulation, oxidative modification, and a pro-inflammatory cascade that promotes foam cell formation and plaque development [10]. Consequently, elevated apoB levels not only reflect an increased atherogenic burden but also play a direct mechanistic role in the initiation and progression of atherosclerosis. Even though LDL-C and apoB levels are highly correlated, more and more research indicates that apoB may be a better predictor for CV risk [11]. In 1980, Sniderman et al. concluded that apoB levels were more efficient in predicting the presence of coronary atherosclerosis compared to other lipid biomarkers [12]. In a recent analysis, which included over 3400 patients, apoB was strongly associated with coronary disease, and, along with other non-classical lipoprotein biomarkers, apoB added value to the standard lipid panel when it came to CV risk [13]. Apolipoprotein B levels may also correlate to atherosclerotic plaque stability and composition; in a very recent intravascular imaging study, Fujino et al. observed that apoB levels < 65 mg/dL were linked to a decreased plaque vulnerability [14].

In addition to apoB, non-high-density lipoprotein cholesterol (non-HDL-C) has emerged as a robust and widely available marker for assessing atherogenic risk. Similarly to apoB, non-HDL-C is a measure of all atherogenic particles, including IDL, LDL, Lp(a), and triglyceride-rich lipoproteins, with a worth-mentioning advantage: it can be easily calculated, using the standard lipid profile, by deducting HDL-C from the total cholesterol [15]. In the context of insulin resistance or hypertriglyceridemia, LDL-C can underestimate the CV risk, as triglyceride-rich particles are causally implicated in atherothrombosis. For this reason, there is a growing belief that non-HDL-C may be a better indicator for CV risk than LDL-C [16]. When it comes to international guidelines, non-HDL-C is generally recognized as a secondary therapeutic target, the 2019 ESC/EAS dyslipidemia guidelines recommending using this biomarker mainly in patients with diabetes, obesity, and hypertriglyceridemia [2]. Similar positions can be seen from the CCS and American Heart Association (AHA), which recommend using non-HDL-C as a risk-enhancing factor [3,17]. In a large-scale meta-analysis that included over 233,400 subjects and over 22,900 events, non-HDL-C was the stronger predictor of CV risk when compared to LDL-C, but was still not as reliable as apoB [18]. Studies also show that non-HDL-C may also be a better indicator of coronary atherosclerosis. In a prospective trial, which included over 1700 patients, Zhang et al. observed a stronger correlation between non-HDL-C and the Gensini score when compared to LDL-C, suggesting a superior predictive value for CAD severity [19]. Similarly, in a study using coronary computed tomography angiography, elevated non-HDL-C was associated with both the presence and the extent of non-calcified coronary plaque, whereas LDL-C was not, thereby linking non-HDL-C more robustly to vulnerable plaque phenotypes [20].

Our study aims to investigate the predictive value of apoB and the LDL-C/apoB ratio for both the presence and the severity of CAD and to compare it with standard lipid biomarkers, including total cholesterol, LDL-C, HDL-C, triglycerides, and non-HDL-C.

## 2. Materials and Methods

### 2.1. Study Design, Patient Enrollment, and Investigations

**Figure 1:**
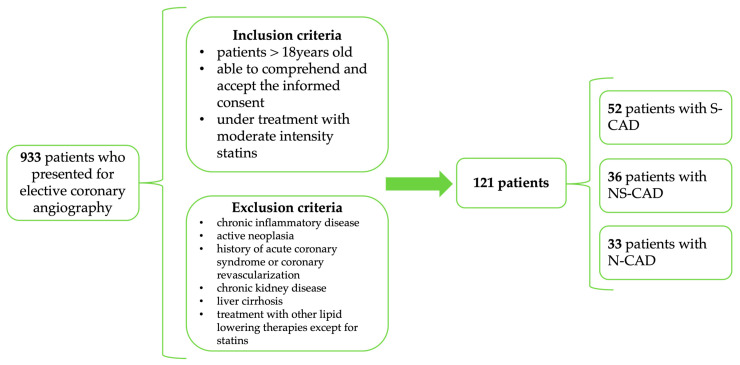
Study design (inclusion and exclusion criteria).

We performed a prospective case–control study that included 121 patients who presented to our hospital for elective coronarography between January 2024 and January 2025. We consecutively enrolled patients referred for elective invasive coronary angiography who were ≥18 years old, able to comprehend and accept an informed consent, who were under treatment with moderate intensity statins (Atorvastatin 10/20 mg or Rosuvastatin 10 mg), and who did not meet any of the following exclusion criteria: chronic inflammatory disease, active neoplasia, chronic kidney disease (eGFR < 30 mL/min/1.73 m^2^), under treatment with other lipid-lowering therapies, except for statins, or liver cirrhosis (Figure 1). Patients with prior coronary revascularization or history of acute coronary syndrome (ACS) were not considered for enrollment, to ensure a homogenous primary prevention cohort. The indication for coronary angiography was previously set, according to the available European Society of Cardiology (ESC) guidelines.

Clinical and paraclinical data were gathered for each participant. Data concerning gender, age, body mass index (BMI), smoking status, and comorbidities [hypertension (HTA), atrial fibrillation (AF), diabetes mellitus (DM), chronic kidney disease (CKD) with eGFR >30 mL/min/1.73 m^2^, chronic pulmonary obstructive disease (COPD), transient ischemic attack (TIA), or stroke] were noted for every patient. We also performed standard transthoracic echocardiography using a GE VIVID™ V7 (General Electric, Boston, CA, USA) ultrasound device, which assessed the general morphofunctional cardiac characteristics. The coronarography was performed using an Azurion 7 Philips Image Guided Therapy System (Philips Healthcare, Best, The Netherlands), and Fraction Flow Reserve (FFR) was used in selected cases. The severity of coronary stenosis was measured using the Quantitative Coronary Analysis (QCA) software. Although inter-observer variability was not formally addressed, all measurements were performed by experienced interventional cardiologists following standardized QCA protocols, which minimized measurement variability. The coronary atherosclerotic burden was quantified using the Gensini score, which provides a semiquantitative assessment of the extent and functional significance of coronary stenosis. For each coronary plaque, a severity score was assigned according to the degree of luminal diameter reduction: 1 point for 1–25% stenosis, 2 points for 26–50% stenosis, 4 points for 51–75% stenosis, 8 points for 76–90% stenosis, 16 points for 91–99% stenosis, and 32 points for complete occlusion. Each lesion severity score was then multiplied by a weighting factor that reflects the functional importance of the myocardial territory supplied by the affected segment: 5 for the left main coronary artery, 2.5 for the proximal left anterior descending (LAD) and proximal circumflex arteries (LCX), 1.5 for the mid LAD, 1 for the distal LAD, right coronary artery (RCA), posterolateral, and obtuse marginal branches, and 0.5 for other smaller branches. The final Gensini score for each patient was obtained by summing the weighted scores across all coronary segments.

Two small samples of venous blood were collected from each enrolled patient. One of them was used for routine blood work [complete blood count, total cholesterol, LDL-C, HDL-C, triglycerides, alanine aspartate transferase (AST), alanine aminotransferase (ALT), serum creatinine, ionogram] and the other one was centrifuged at 2000 rpm for 20 min. The obtained serum was stored at −80 °C, for no longer than 2 months, until apoB analysis was performed using an immunoturbidimetric assay.

### 2.2. Statistical Analysis

Statistical analysis was performed using the SPSS v26.0 software package (SPSS Inc., Chicago, IL, USA). For all analyses that we performed, the threshold for statistical significance was a *p*-value < 0.05. Standard descriptive statistics parameters (mean, standard deviation, minimum, maximum, and median values) were calculated for numerical data. Differences between arithmetical means of two samples were tested using Student’s *t*-test, after testing for normal distribution using the Kolmogorov–Smirnov test. For comparisons between multiple groups of samples, the ANOVA test was performed. In cases of statistical significance, a post hoc Tukey correction was used to adjust for multiple comparisons. For categorical variables, we used frequencies and percentages to express descriptive data, and the differences across the studied groups were tested using the Chi-squared test.

In order to assess the correlations between studied biomarkers and the Gensini score, we used Spearman’s r (r), with corresponding *p*-values and 95% confidence intervals. To further explore the relationship between correlated biomarkers, we used univariate linear regression models, where the different biomarkers were used as the independent variable and the Gensini score as the dependent variable. Before performing the linear regression analysis, we applied a natural logarithmic transformation using the formula ln(Gensini + 1). Adding a constant of 1 improved the symmetry of the variable, considering the relatively large proportion of patients with N-CAD (Gensini = 0). Subsequently, multivariate linear regression models were constructed to adjust for traditional risk factors. The coefficient of determination (R^2^) was reported to quantify the proportion of variability in the Gensini score explained by our models.

To explore LDL-apoB discordance, we calculated the residual apoB, a variable that captures the portion of apoB which is not explained by LDL-C. To do so, we performed a linear regression analysis using apoB as the dependent variable and LDL-C as the independent variable and saved the residual values. A positive residual apoB reflected apoB concentrations higher than expected for a given LDL-C level, whereas a negative residual apoB reflected lower than expected concentrations. Subsequently, we performed logistic regression models using residual apoB as the independent variable.

Receiver operating characteristic (ROC) curve analysis was performed to test the diagnostic performance of the studied biomarkers in detecting coronary artery disease and the areas under the curve (AUCs) were used to make a comparison between them.

### 2.3. Ethics

Our prospective study was conducted according to the ethical principles stated in the Declaration of Helsinki (revised in 2013). At the moment of enrollment, every patient signed a standard informed consent. All processed data were anonymous, and the entire research was approved by Ethics Committees of both University of Medicine and Pharmacy “Grigore T. Popa” Iași (no. 376/7 January 2024) and of the St. Spiridon Emergency Clinical Hospital (no. 82/25 September 2023).

## 3. Results

### 3.1. Baseline Characteristics

We enrolled 121 patients who were further divided into three subgroups: 52 patients with S-CAD (significant coronary artery disease, defined as stenosis ≥ 50% for left main, or stenosis ≥ 70% for any other coronary artery), 36 patients with NS-CAD (non-significant coronary artery disease), and 33 patients with N-CAD (no coronary artery disease, defined as the absence of atherosclerotic plaques, visible at standard coronarographic examination).

Table 1 summarizes the general demographic characteristics and paraclinical findings of the patients included in our study. There was no statistically significant difference in terms of non-modifiable CV risks factors between the three groups. However, we observed a higher prevalence of the male gender (*p* = 0.390) and smoking status (*p* = 0.722) in NS-CAD and S-CAD groups compared to N-CAD. Other CV risk factors were more prevalent in S-CAD or NS-CAD groups compared to N-CAD. The overall prevalence of AF was 28.9% and, despite the lack of statistical significance (*p* = 0.388), there was a noteworthy difference between the NS-CAD (30.5%) and S-CAD (30.7%) when compared to the N-CAD group (18.1%). Diabetes was more prevalent only in S-CAD (30.7%).

The echocardiographic assessment did not reveal any significant differences between the three studied groups (Supplementary Data Table S8). However, both LA- and RA-indexed area mean values gradually increased across the three groups, the difference approaching statistical significance (*p* = 0.065 and *p* = 0.067, respectively). The mean LVEF was 51.49%, with a standard deviation of 11.71%, and no statistically significant difference was observed in the three groups.

### 3.2. Lipid Parameters

In order to evaluate whether any of the lipid biomarkers that we studied were predictive for the presence of CAD, we compared the mean values across the three groups (Table 2). Within the standard lipid profile, the mean values for total cholesterol (TC) (149.37 ± 27.45 vs. 161.12 ± 37.12 vs. 178.86 ± 48.30, *p* = 0.006) and LDL-C (86.40 ± 24.67 vs. 98.44 ± 34.52 vs. 115.53 ± 43.64) increased progressively across the three groups (*p* = 0.003). As for HDL-C, even though the mean values were lower in NS-CAD and S-CAD compared to N-CAD, the difference did not reach statistical significance (*p* = 0.831). Unsurprisingly, non-HDL-C also presented higher mean values in the NS-CAD group (108.36 ± 43.33) and S-CAD group (127.40 ± 49.64) compared to the N-CAD group (93.48 ± 38.10), with a significant *p*-value (0.003).

By far the best performing biomarker was apoB. In our comparative analysis across the three study groups, it demonstrated the most pronounced intergroup variation. The mean apoB concentrations differed significantly (63.21 ± 18.17 vs. 82.44 ± 24.31 vs. 93.17 ± 27.87), with statistical testing confirming a highly robust association (*p* < 0.001). As for the LDL/ApoB ratio, we also registered significant variations (*p* = 0.012) between the three groups; however, the mean values tended to increase in S-CAD when compared to NS-CAD (1.25 ± 0.25 vs. 1.18 ± 0.23).

**Table 2:** Mean values of different lipid biomarkers across the studied groups.

Parameter	Overall	N-CAD	NS-CAD	S-CAD	*p*-Value
TC (mg/dL)	165.81 ± 41.97	149.37 ± 27.45	161.12 ± 37.12	178.86 ± 48.30	**0.006**
LDL-C (mg/dL)	102.65 ± 38.38	86.40 ± 24.67	98.44 ± 34.52	115.53 ± 43.64	**0.003**
HDL-C (mg/dL)	46.82 ± 12.97	48.14 ± 14.93	46.38 ± 9.50	46.36 ± 13.97	0.831
Triglycerides (mg/dL)	107.48 ± 50.23	100.37 ± 47.22	107.12 ± 46.83	112.08 ± 54.58	0.606
Non-HDL-C (mg/dL)	112.49 ± 46.72	93.48 ± 38.10	108.36 ± 43.33	127.40 ± 49.64	**0.003**
ApoB (mg/dL)	82.34 ± 27.29	63.21 ± 18.17	82.44 ± 24.31	93.17 ± 27.87	**0.000**
LDL/ApoB	1.27 ± 0.32	1.41 ± 0.45	1.18 ± 0.23	1.25 ± 0.25	**0.012**

Statistically significant *p*-values are highlighted in bold.

In order to identify specific pairwise differences between the three groups, we performed a post hoc Tukey analysis (Supplementary Table S1). All parameters included in the Tukey analysis showed a normal distribution pattern. Patients in the N-CAD group had significantly lower apoB levels compared to both NS-CAD (*p* = 0.002) and S-CAD (*p* < 0.001). Between NS-CAD and S-CAD, we observed a mean difference of 10.6 mg/dL; however, it did not reach the statistical significance threshold (*p* = 0.103). As for TC, LDL-C, and non-HDL-C, statistical significance was observed only between the N-CAD and S-CAD groups, whereas the LDL/apoB ratio differed significantly only between N-CAD and NS-CAD.

### 3.3. Correlation with Gensini Score

In Table 3 and Supplementary Table S2, we presented Spearman’s correlation analysis between the studied biomarkers and the Gensini score. As expected, apoB positively correlated with LDL-C, TC, triglycerides, and non-HDL-C. Regarding the Gensini score, our analysis revealed significant associations with several lipid biomarkers. Based on the correlation coefficient (r), apoB demonstrated the strongest positive correlation with CAD severity (r = 0.430, *p* < 0.001), followed by non-HDL-C (r = 0.285, *p* = 0.002) and LDL-C (r = 0.268, *p* = 0.004). A significant correlation with the Gensini score was not observed for triglycerides, HDL-C, or the LDL/ApoB ratio.

### 3.4. Diagnostic Performance

**Figure 2:**
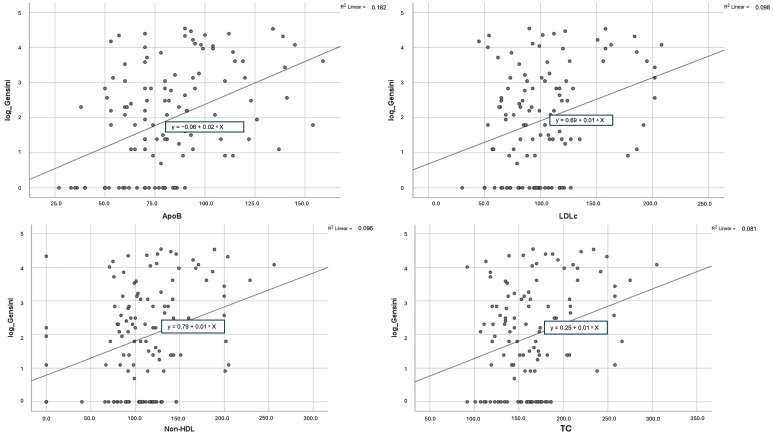
Scatterplots expressing the correlation between apoB, non-HDL-C, LDL-C, and TC and log-transformed Gensini score.

To test the diagnostic performance of the biomarkers that presented significant mean differences between the studied groups and also a significant correlation with the Gensini score, we performed univariate linear regression (Table 4). One more time, apoB showed the strongest association with the severity of CAD (B = 0.024, 95% CI: 0.015–0.034, β = 0.427, R^2^ = 0.182, *p* < 0.001), as each 1 mg/dL increase in apoB was linked to a 0.024-point rise in the log-transformed Gensini score. Non-HDL-C (B = 0.010, 95% CI: 0.004–0.016, β = 0.310, R^2^ = 0.096, *p* = 0.001), LDL-C (B = 0.012, 95% CI: 0.005–0.019, β = 0.312, R^2^ = 0.098, *p* = 0.001), and TC (B = 0.010, 95% CI: 0.004–0.017, β = 0.285, R^2^ = 0.081, *p* = 0.002) also showed a significant correlation with the atherosclerotic burden; however, the association was weaker (Figure 2). As for the LDL/ApoB ratio, the linear regression analysis failed to reach statistical significance.

Even after adjusting for other traditional CV risk factors (age, sex, smoking status, DM, BMI, blood pressure, AF, and eGFR), apoB remained an independent predictor for CAD severity, along with DM and age (Table 5).

Apolipoprotein B also proved superior in detecting significant CAD (Table 6 and Supplementary Table S3). For comparability, continuous variables were standardized. In the bivariate logistic regression, each standard deviation (SD) increase in apoB corresponded to a 2.386-fold increase in the odds of significant coronary stenosis. Moreover, apoB was the only biomarker able to predict left main disease, with an OR of 2.433 per SD increase (Table 7A and Supplementary Table S4). In order to eliminate the influences of confounding CV risk factors, we performed a multivariate logistic regression, which included age, female sex, smoking status, DM, BMI, AF, hypertension, and eGFR (Table 7B and Supplementary Table S5). The resulting model explained 29.9% of the variances in the diagnosis of significant CAD (Nagelkerke R^2^ = 0.299) and 41.6% in the diagnosis of left main disease (Nagelkerke R^2^ = 0.416), apoB remaining significant in both cases (OR 2.506, 95% CI 1.458–4.308, *p* = 0.000; OR 4.792, 95% CI 1.574–14.585, *p* = 0.006, respectively). Interestingly, apoB alone was not associated with three vessel disease in the bivariate logistic regression. However, after a multivariate adjustment, apoB became significant (OR 1.458, 95% CI 0.848–2.504, *p* = 0.045).

The LDL/ApoB ratio did not show a statistically significant prediction power for S-CAD (Table 6). However, when testing for the presence of coronary atherosclerosis, the LDL/ApoB ratio was negatively associated with the disease (B = −0.593, *p* = 0.010, OR 0.553, 95% CI: 0.352–0.867, R^2^ = 0.096), suggesting that a lower ratio is linked to a greater likelihood of coronary disease. In contrast, apoB consistently demonstrated a markedly stronger predictive performance and statistical significance (B = 1.533, *p* < 0.001, OR 4.634, 95% CI: 2.307–9.309, R^2^ = 0.311) (Table 8 and Supplementary Table S6).

Finally, to test the predictive power of apoB independent of LDL-C, we performed a bivariate logistic regression, using residual apoB. As shown in Table 9 and Supplementary Table S7, even though the model’s explanatory power decreased as compared to the initial apoB analysis (Nagelkerke R^2^ 0.105 vs. 0.311), it remained statistically significant (*p* = 0.001, OR 5.222, 95% CI: 1.932–14.116). For significant CAD, the logistic model failed to reach statistical significance (*p* = 0.089, OR 1.923, CI: 0.907–4.080, Nagelkerke R^2^ 0.033).

## 4. Discussion

Our study aimed to evaluate the role of apolipoprotein B (apoB) as a biomarker for chronic coronary syndrome (CCS) and to compare its diagnostic efficiency with conventional lipid biomarkers. We not only evaluated the diagnostic value of the studied biomarkers in relation to the presence of CAD, but also their relationship with the severity of the disease, quantified using the Gensini score. We included a total of 121 patients who presented for elective coronarography in an Eastern European County Hospital, our results complementing the limited evidence from Eastern and Central European centers.

The general characteristics of the patients were similar across the three groups. The mean age in our cohort was 64.2 ± 9.3 years old (y.o), close to the average seen in larger European CAD cohorts [21]. Additionally, the male gender was better represented in all three groups, which is an expected finding given the higher CV risk in men [22]. Substantial differences regarding classical CV risk factors were observed between the three studied groups; however, statistical significance was not reached. We observed similar rates of AF in NS-CAD and S-CAD (30.5% vs. 30.7%), which were significantly higher compared to the N-CAD group (18.1%). This finding supports the theory that there is a bidirectional relationship between AF and CAD. Ischemia-induced myocardial remodeling can cause electrical changes that promote AF, whereas the hemodynamic stress, diastolic dysfunction, and increased oxygen consumption caused by this arrhythmia can worsen the effects of CAD [23]. Moreover, the similar rates of AF between the two groups with CAD may be explained by the fact that even non-significant coronary atherosclerosis may induce atrial ischemia. In their study, Kornej et al. concluded that coronary artery sclerosis (stenosis < 75%) was associated with a higher risk of AF [24].

In our comparative analysis, we observed that the mean values of a portion of traditional lipid markers (TC, LDL-C, and non-HDL-C) and apoB increased progressively and significantly across the three studied groups; however, apoB proved to be a more sensitive indicator of coronary atherosclerosis. Unlike traditional lipid biomarkers, apoB levels differed significantly not only between N-CAD and S-CAD, but also between N-CAD and NS-CAD, indicating a possible indicator even for mild, non-obstructive atherosclerotic disease. This superior discrimination power for atherosclerosis that characterized apoB in our analysis may be attributed to the fact that apoB directly reflects the number of all atherogenic particles [25]. Circulating atherogenic lipoproteins, including LDL, IDL, VLDL, and Lp(a), contain a single apoB molecule. On the other hand, biomarkers like LDL-C or non-HDL-C measure the total cholesterol mass in atherogenic particles, which is highly variable. Knowing that, the total CV risk may be underestimated in patients with a large number of cholesterol-depleted lipoproteins but with an increased apoB [26]. This aligns with the theory that the “quality” and not “quantity” of LDL is also important, as smaller, but denser in cholesterol, LDL particles may be better associated with coronary atherosclerosis. In our study, we also evaluated the role of the LDL-C/apoB ratio, which is a measure of the average cholesterol content in LDL molecules. As demonstrated in previous studies, a lower ratio is associated with an increased CV risk, due to the higher atherogenicity of LDL particles [27]. We did not register any notable differences between NS-CAD and S-CAD; however, the LDL-C/apoB ratio was lower among patients with coronary atherosclerosis. The fact that our study did not register a progressive decrease in the LDL-C/apoB ratio across the three groups does not fully contradict the theory of “LDL quality”, as atherosclerosis is a complex process in which cholesterol is only one of the main players. Moreover, in both NS-CAD and S-CAD, the LDL-C/apoB mean ratios were very close to the threshold value associated with an increased risk of coronary atherosclerosis, as suggested in the available literature [28].

The diagnostic superiority of apoB extended beyond group mean differences, as it also demonstrated the strongest correlation with CAD severity, as expressed by the Gensini score. In our study, apoB showed a moderate correlation with the Gensini score (r = 0.43, *p* < 0.001), which was markedly higher compared to LDL-C, TC, or non-HDL-C, all of which presented only a weak correlation (r = 0.26, r = 0.23, and r = 0.28, respectively). This aligns with previous data, as Yaseen et al. found a significant correlation between apoB and the Gensini score (r = 0.22), even though the study was conducted on patients with ACS [29]. Similarly, Haidari et al. conducted a prospective analysis of normolipidemic patients and found that apoB was the best predictor of CAD presence. They also reported a significant, but lower correlation than in our cohort between this biomarker and the Gensini score (r = 0.22) [30]. None of these studies reported the correlation coefficient for LDL-C or non-HDL-C for comparison. Moreover, apoB also performed better in univariate linear regression, where the log-transformed Gensini score was used as the dependent variable. In our analysis, for each 1 mg/dL increase in serum apoB, the log-transformed Gensini score increased by 0.024 compared to LDL-C or non-HDL-C, which registered increases in Gensini of only 0.012 and 0.010, respectively. ApoB remained significant, even after adjusting for traditional CV risk factors. This suggests that it may be used as an indicator for the extension of CAD, as higher serum levels track closely with more extensive and severe coronary atherosclerosis. Huynh et al. reported an increase in the Gensini score by 0.78 points for each 1 mg/dL increase in apoB. In their analysis, the Gensini score was not log-transformed and patients under lipid-lowering therapy were excluded, which explains the higher impact of apoB in their paper [31].

Contrary to LDL-C, non-HDL-C aggregates the cholesterol content of all apoB-containing lipoproteins and generally correlates well with serum apoB, being considered a surrogate for this apolipoprotein [32]. In our cohort, non-HDL-C behaved similarly to LDL-C in most analyses, while apoB showed a stronger association with the presence and the severity of CAD. This happened despite the strong correlation between non-HDL-C and apoB (r = 0.734, *p* < 0.001). Our finding might be explained by apoB discordance, a situation where apoB is high relative to LDL-C or TC. Up to 20% of individuals manifest this phenomenon, often in the presence of metabolic syndrome or diabetes [5]. Statin use also proved to be responsible for such a discordance, as shown by Johannesen et al. Patients under statin treatment who presented with elevated apoB, but not elevated non-HDL-C, presented a higher risk for CV mortality [33].

Moreover, the logistic regression analysis further supported the superiority of apoB in detecting the presence of CAD. Apolipoprotein B was not only superior to standard lipid biomarkers in predicting significant CAD, but it was also the only biomarker capable of predicting left main stenosis. Each SD increase in apoB corresponded to an OR of 2.386 for significant coronary stenosis and an OR of 2.433 for left main atherosclerosis. Left main disease is particularly relevant in CAD diagnosis, due to its higher mortality and association with multivessel disease [34]. Our results align with those reported in the available literature [35,36] and may suggest that apoB is a more accurate indicator of CV risk and mortality. In a meta-analysis which compared apoB, LDL-C, and non-HDL-C, Sniderman et al. demonstrated that apoB was the most potent marker for CV risk, suggesting that an apoB-based prevention strategy may reduce 800,000 more coronary events than the LDL-C based strategy [18]. In a prospective study, conducted on a UK biobank cohort which included over 389,000 patients, apoB had a complementary role to SCORE2 thresholds in identifying patients at low risk. Even though integrating apoB in SCORE2 did not improve the model’s efficiency, participants with both a low SCORE2 and low apoB had lower CV events compared to those with a low SCORE2 alone [37].

Another noteworthy finding in our study is related to the LDL/ApoB ratio. It is considered that the atherogenicity of LDL is not only determined by the total mass of circulating cholesterol, but also by the lipoprotein’s biochemical characteristics, especially its mass. Smaller LDL particles are considered more atherogenic, as they more easily reach the subendothelial space and have higher retention rates in the arteries [38]. As LDL particle size is reasonably challenging to measure, the LDL/ApoB ratio was introduced as its surrogate. A lower LDL/ApoB ratio proved to be associated with an increased risk of adverse CV events, and also with the presence and severity of CAD [27,28]. In our study, even though patients with NS-CAD and S-CAD presented lower LDL/ApoB ratios when compared to N-CAD (1.18 ± 0.23 vs. 1.25 ± 0.25 vs. 1.41 ± 0.45), the correlation between this biomarker and the Gensini score was very weak and failed to reach statistical significance (r = −0.073, *p* = 0.440). The logistic regression analysis showed consistent results, as the LDL/ApoB ratio was negatively associated with the presence of coronary atherosclerosis (B = −0.593, *p* = 0.010, OR 0.553, 95% CI: 0.352–0.867, R^2^ = 0.096); however, it showed a limited predictive power for significant CAD. Our results support the LDL/ApoB ratio as a negative predictive factor for CAD initiation, but not for its progression. In other words, smaller LDL particles may be able to initiate plaque formation, but not necessarily promote its progression over time, as atherosclerosis is a multifactorial process. Another explanation for our finding may be statin use. Statins typically lower LDL-C significantly, but they may not reduce the LDL particle number proportionately. In fact, moderate-dose statin therapy often leaves a greater proportion of small, dense LDL particles [39]. Consequently, in a satin-treated cohort, the LDL-C/apoB ratio may tend to cluster at lower values, potentially canceling any gradient that would correlate with CAD severity.

In our study, as expected, apoB and LDL-C were highly correlated (r = 0.768, *p* = 0.000). This naturally raised the question of whether the strong association between apoB and CAD was due to apolipoprotein’s own intrinsic effect or due to LDL-C’s influence. Consequently, we calculated residual apoB, using linear regression analysis, by subtracting the predicted apoB value from the measured one. The logistic regression analysis showed a significant association between residual apoB and the presence of coronary atherosclerosis; however, statistical significance was not reached for S-CAD (*p* = 0.089). Given the small representation of patients with hemodynamically significant CAD, this negative result should be interpreted with caution. Extensive evidence supports the link between a high residual apoB and an increased risk of MACE [40,41]. However, papers evaluating the association between residual apoB and imaging-confirmed CAD are relatively scarce. In the few studies that we could find, discordantly high apoB was predictive for subclinical atherosclerotic CV disease and a higher coronary artery calcium score (CAC score) [42]. Studies quantifying coronary stenosis, beyond the CAC score, are even more limited. In the only study that we could find, residual apoB correlated with both coronary atherosclerosis and its burden, patients being evaluated via computed tomography angiography [43]. Lastly, in another prospective study which included over 14,000 patients, residual apoB was associated with CAC score progression over a follow-up period of 5 years [44]. Given these results, the hypothesis that residual apoB is associated only with the initial phases of coronary atherosclerosis, but not with its progression, is not plausible. Our non-significant findings most likely reflect a limited statistical power generated by the small S-CAD cohort rather than a true absence of association. Future prospective studies with larger cohorts are needed to clarify the role of residual apoB in CAD development.

Only a few studies which included patients from Eastern European countries examined the relationship between apoB and atherosclerotic disease, and even fewer assessed CAD. In a Polish study, Owczarek et al. showed that apoB levels were higher in women with significant CAD, but not in men. The study was retrospective and did not exclude patients with a history of ACS or revascularization. Moreover, there is no data regarding statin use in the studied population [45]. Melnychuk et al. showed in their analysis that patients with CAD had higher levels of apoB compared to the control group, yet apoB was not the main biomarker evaluated, and the study did not include QCA lesion quantification or specific information about CAD burden [46]. We found additional information in a Romanian study from the SEPHAR registry, in which apoB was associated with significant carotid atherosclerosis and other classical markers of clinical and subclinical CV disease. Also, myocardial infarction and angina pectoris were more prevalent in the high apoB group; however, there was no information regarding coronary anatomy [47]. In contrast to these prior studies, our work provides more detailed insights into the association between apoB and chronic CAD, in a statin-treated Eastern European cohort undergoing elective invasive coronary angiography.

### Study Limitations

Several limitations should be acknowledged when interpreting the results of this study. By far the most significant limitation is the unicentric design of our study, which limits external validity. As illustrated by region-calibrated risk tools (e.g., SCORE2), risk stratification and model calibration vary across different regions. In other words, the diagnostic performance of the studied biomarkers may also be sensitive to regional differences in baseline CAD prevalence, prevention and treatment patterns, and population structure. Additionally, our cohort is relatively small, which contributed to the lack of statistical significance for several CV risk factors (most notably the LDL/apoB ratio, residual apoB, age, sex distribution, smoking status, and TG levels), despite substantial differences between groups. This situation mainly occurred due to an increased prevalence of statin use, which limited the patient pool significantly.

All the patients in our cohort were under moderate-intensity statin therapy. Consequently, the measured values for all lipid parameters, including apoB, mainly reflect the post-treatment residual atherogenic risk rather than the baseline risk. Since atherosclerosis is highly influenced by the total time of exposure to a certain level of atherogenic particles, pre-statin apoB levels might have offered a greater accuracy and may have better captured the true magnitude of the risk inflicted by this lipoprotein. However, pre-treatment data were not available, and this limitation must be considered when interpreting the results from our study.

Lastly, the data we presented corresponded to a single determination of the studied biomarkers. Therefore, we were unable to investigate the long-term impact on CV risk or CAD progression, which would have provided prognostic information. Also, serial measurements would allow us to evaluate the effect of lipid-lowering agents on apoB levels and investigate the outcomes of an apoB-centered approach for CV risk management.

## 5. Conclusions

In our pilot study, apoB consistently outperformed traditional lipid biomarkers in diagnosing CAD. It showed the strongest predictive power for CAD, independent of LDL-C, as demonstrated by the residual apoB analysis. Apolipoprotein B was also the only biomarker independently associated with left main atherosclerosis, also showing a strongest association with the Gensini score.

Consequently, our results reinforce the growing evidence that apoB is a more valuable marker of atherogenicity and expand the limited evidence available from Eastern European countries. However, as a single-center observational study and given the relatively small cohort, our results are preliminary; larger prospective cohorts are needed to validate whether an apoB-based management of patients suspected of having CAD can better predict or improve clinical outcomes.

## Figures and Tables

**Table 1:** Demographic, laboratory, and echocardiographic findings of the patients included in the study.

Parameter	Overall	N-CAD	NS-CAD	S-CAD	*p*-Value
Age (years)	64.2 ± 9.3	61.9 ± 10.2	63.8 ± 8.3	66 ± 9.33	0.192
Male gender (%)	63.6%	54.5%	63.9%	69.2%	0.390
Smoking (%)	56.2%	51.5%	61.1%	55.8%	0.722
Pack-years	18.19 ± 22.68	14.30 ± 17.85	21.25 ± 28.14	18.54 ± 21.22	0.445
BMI (kg/m^2^)	30.17 ± 5.70	30.53 ± 7.35	30.94 ± 4.37	29.42 ± 5.34	0.436
AF (%)	28.9%	18.1%	30.5%	30.7%	0.388
DM (%)	24.8%	24.2%	15.7%	30.7%	0.481
CKD (%)	21%	15.2%	25%	22%	0.589
PAD (%)	10.7%	6.1%	5.6%	17.3%	0.129
COPD (%)	9.1%	0.3%	11.1%	13.5%	0.096
Stroke/TIA (%)	9.9%	6.1%	11.1%	11.5%	0.682
Biological parameters					
Glycaemia (mg/dL)	110.15 ± 29.83	115.04 ± 37.81	105.62 ± 21.42	110.71 ± 30.25	0.477
Ly/neut	0.49 ± 0.24	0.47 ± 0.17	0.5 ± 0.26	0.5 ± 0.26	0.778
AST (U/L)	24.45 ± 10.79	23.29 ± 5.85	26.81 ± 16.58	23.41 ± 6.92	0.331
ALT (U/L)	24.74 ± 12.97	23.14 ± 8.09	26.82 ± 17.89	24.06 ± 10.69	0.522
GGT (U/L)	49.07 ± 57.57	40.38 ± 26.68	69.95 ± 87.15	40.17 ± 40.74	0.127
Creatinine (mg/dL)	0.88 ± 0.29	0.88 ± 0.31	0.89 ± 0.26	0.87 ± 0.30	0.952
eGFR (mL/min/1.73 m^2^)	86.43 ± 18.35	86.51 ± 20.56	85.80 ± 18.85	86.86 ± 16.81	0.968

BMI: body mass index; AF: atrial fibrillation; DM: diabetes mellitus; CKD: chronic kidney disease; PAD: peripheral artery disease; COPD: chronic obstructive pulmonary disease; TIA: transient ischemic stroke; Ly/neut: lymphocyte-to-neutrophil ratio.

**Table 3:** Spearmen’s correlation of different lipid biomarkers and Gensini score.

		Gensini Score	LDL-C	HDL-C	Non-HDL-C	LDL/ApoB
Gensini Score	r	1.000	0.268 **	0.084	0.285 **	0.073
	*p*-value (95% CI)		0.004 (0.074–0.441)	0.378 (−0.253–0.131)	0.002 (−0.242–0.158)	0.440 (−0.242–0.158)
	*N*	121	121	121	121	121
ApoB	r	0.430 **	0.768 **	0.152	0.734 **	0.087
	*p*-value (95% CI)	0.000 (0.202–0.543)	0.000 (0.658–0.847)	0.109 (−0.011–0.350)	0.000 (0.711–0.877)	0.360 (−0.263–0.150)
	*N*	121	121	121	121	121

**: Correlation is significant at the 0.01 level (2-tailed); r: correlation coefficient; *N*: number of values.

**Table 4:** Univariate linear regression analysis of lipid biomarkers with log-transformed Gensini score as dependent variable.

Biomarker	B	Std. Error	Standardized β	95% CI for B	R^2^	*p*-Value
LDL-C	0.012	0.004	0.312	0.005–0.019	0.098	**0.001**
Non-HDL-C	0.010	0.003	0.310	0.004–0.016	0.096	**0.001**
ApoB	0.024	0.005	0.427	0.015–0.034	0.182	**<0.001**
TC	0.010	0.003	0.285	0.004–0.017	0.081	**0.002**
LDL/ApoB	−0.752	0.440	−0.160	−1.624–0.120	0.026	0.090

Statistically significant *p*-values are highlighted in bold.

**Table 5:** Multivariate linear regression model of apoB and traditional CV risk factors (age, sex, smoking status, DM, BMI, hypertension, AF, eGFR) with log-transformed Gensini score as dependent variable.

Biomarker	B	Std. Error	Standardized β	95% CI for B	Model R^2^	*p*-Value
ApoB	0.024	0.005	0.421	0.015–0.034	0.313	**<0.001**
Constant	−4.671	2.110	-	−8.859–−0.484		**0.029**

Statistically significant *p*-values are highlighted in bold.

**Table 6:** Bivariate logistic regression: selected lipid biomarkers as predictors for significant coronary artery disease.

Biomarker	OR (95% CI)	*p*-Value	Nagelkerke R^2^
ApoB (SD)	2.386 (1.52–3.75)	0.000	0.185
LDL-C (SD)	1.905 (1.24–2.92)	0.001	0.115
LDL/ApoB (SD)	0.865 (0.588–1.271)	0.460	0.007
Non-HDL-C (SD)	1.884 (1.23–2.9)	0.004	0.106
TC (SD)	1.819 (1.2–2.76)	0.003	0.101

**Table 7:** (**A**) Bivariate logistic regression: standardized apoB as predictor for left main atherosclerosis and three vessel disease. (**B**) Multivariate logistic regression model of standardized apoB and traditional CV risk factors (age, sex, smoking status, DM, BMI, AF, hypertension, and eGFR).

Outcome	OR (95% CI)	*p*-Value	Nagelkerke R^2^
(**A**) Simple bivariate analysis			
Left main disease	2.43 (1.38–4.30)	**0.002**	0.160
Three vessel disease	1.52 (0.98–2.37)	0.063	0.045
(**B**) Multivariate logistic regression			
Significant CAD	2.51 (1.46–4.31)	**0.000**	0.299
Left main disease	1.46 (0.85–2.5)	**0.045**	0.221
Three vessel disease	4.79 (1.57–14.59)	**0.006**	0.416

Statistically significant *p*-values are highlighted in bold.

**Table 8:** Bivariate logistic regression: comparison between apoB and LDL/ApoB ratio as predictors for coronary atherosclerosis.

Biomarker	OR (95% CI)	*p*-Value	Nagelkerke R^2^
ApoB (SD)	4.64 (2.31–9.31)	0.000	0.311
LDL/ApoB ratio (SD)	0.55 (0.35–0.87)	0.010	0.096

**Table 9:** Bivariate logistic regression: residual apoB as predictor for coronary atherosclerosis and significant CAD.

Outcome	OR (95% CI)	*p*-Value	Nagelkerke R^2^
Coronary atherosclerosis	5.22 (1.93–14.12)	0.001	0.105
Significant CAD	1.92 (0.91–4.08)	0.089	0.033

## Data Availability

All data presented in this study are available within the article. The first author has all data used in this study.

## Supplementary Materials

The following supporting information can be downloaded at https://www.mdpi.com/article/10.3390/diagnostics15233002/s1. Table S1. Post-hoc Tukey analysis for lipid biomarkers. Table S2. Spearmen’s correlation of different lipid biomarkers and Gensini score. Table S3. Bivariate logistic regression: selected lipid biomarkers as predictors for significant coronary artery disease. Table S4. Bivariate logistic regression: standardized apoB as predictor for left main atherosclerosis and three vessels disease. Table S5. Multivariate logistic regression model of standardized apoB and traditional CV risk factors (age, sex, smoking status, DM, BMI, AF, hypertension and eGFR). Table S6. Bivariate logistic regression: comparison between apoB and LDL/ApoB ratio as predictors for coronary atherosclerosis. Table S7. Bivariate logistic regression: residual apoB as predictor for coronary atherosclerosis and significant CAD. Table S8. Echocardiographic findings of the patients included in the study. Figure S1. ROC curve showing the association between studied biomarkers and coronary atherosclerosis and significant CAD.
