# Supplementary material for: Moving Beyond LDL-C and Non-HDL-C: Apolipoprotein B as the Stronger Lipid-Related Predictor of Coronary Artery Disease in Statin-Treated Patients
Source: Diagnostics (Basel). 2025 Nov 26;15(23):3002. doi: 10.3390/diagnostics15233002 (PMC12691110; doi:10.3390/diagnostics15233002)
Supplement: Supplementary file 1 [file diagnostics-15-03002-s001.zip › diagnostics-3968371-supplementary.pdf]

Tabel S1. Post-hoc Tukey analysis for lipid biomarkers

			Mean Difference	Std. Error	p-value	95% CI	
						Lower bound	Upper bound
**LDL-C**	N-CAD	NS-CAD	-12,04	9,19	0.393	-33.89	9.81
		S-CAD	-29.13*	8.51	0.003	-49.35	-8.90
	S-CAD	N-CAD	29.13*	8.51	0.003	8.90	49.35
		NS-CAD	17.08	8.19	0.098	-2.38	36.56
**TC**	N-CAD	NS-CAD	-11.75	10.13	0.481	-35.82	12.31
		S-CAD	-29.49*	9.34	0.006	-51.68	-7.30
	S-CAD	N-CAD	29.49*	9.34	0.006	7.30	51.68
		NS-CAD	17.74	8.99	0.124	-3.61	39.10
**Triglycerides**	N-CAD	NS-CAD	-6.75	12.63	0.855	-36.77	23.27
		S-CAD	-11.71	11.69	0.577	-39.50	16.07
	S-CAD	N-CAD	11.71	11.69	0.577	-16.07	39.50
		NS-CAD	4.96	11.26	0.899	-21.79	31.72
**Non-HDL-C**	N-CAD	NS-CAD	-14.87	10.82	0.357	-40.56	10.81
		S-CAD	-33.91*	9.99	0.003	-57.64	-10.21
	S-CAD	N-CAD	33.91*	9.99	0.003	10.21	57.64
		NS-CAD	19.04	9.73	0.128	-4.06	42.15
**ApoB**	N-CAD	NS-CAD	-19.68*	5.72	0.002	-33.28	-6.09
		S-CAD	-30.29*	5.29	0.000	-42.84	-17.73
	S-CAD	N-CAD	30.29*	5.29	0.000	17.73	42.84
		NS-CAD	10.60	5.15	0.103	-1.63	22.83
**LDL/ApoB**	N-CAD	NS-CAD	0.23*	0.07	0.011	0.044	0.41
		S-CAD	0.16	0.07	0.057	-0.004	0.33
	S-CAD	N-CAD	-0.16	0.07	0.057	-0.33	0.004
		NS-CAD	0.06	0.06	0.639	-0.102	0.22

Table S2. Spearmen's correlation of different lipid biomarkers and Gensini score

		Gensini Score	LDL-C	HDL-C	TC	Triglycerides	Non-HDL-C	ApoB	LDL/ApoB
Scor Gensini	r	1.000	.268**	-0.084	.232*	0.175	.285**	.430**	-0.073
	P-value		**0.004**	0.378	**0.013**	0.064	**0.002**	**0.000**	0.440
	N	121	121	121	121	121	121	121	121
LDL-C	r	.268**	1.000	0.152	.886**	.204*	.943**	.768**	.511**
	P-value	**0.004**		0.110	**0.000**	0.031	**0.000**	**0.000**	**0.000**
	N	121	121	121	121	121	121	121	121
Non-HDL-C	r	.285**	.943**	0.132	.917**	.335**	1.000	.734**	.426**

	P-value	**0.002**	**0.000**	0.164	**0.000**	**0.000**		**0.000**	**0.000**
	N	121	121	121	121	121	121	121	121
ApoB	r	.430**	.768**	0.152	.753**	.282**	.734**	1.000	-0.087
	P-value	**0.000**	**0.000**	0.109	**0.000**	**0.002**	**0.000**		0.360
	N	121	121	121	121	121	121	121	121
LDL/ApoB	r	-0.073	.511**	0.018	.363**	-0.048	.426**	-0.087	1.000
	P-value	0.440	**0.000**	0.853	**0.000**	0.615	**0.000**	0.360	
	N	121	121	121	121	121	121	121	121

**: Correlation is significant at the 0.01 level (2-tailed); *: Correlation is significant at the 0.05 level (2-tailed); r: correlation coefficient; N: number of values

Table S3. Bivariate logistic regression: selected lipid biomarkers as predictors for significant coronary artery disease

**Biomarker**	**B**	**SE**	**Wald**	**dF**	**OR**	**95% CI for OR**	**p-value**	**Nagelkerke R^2**
**ApoB**								
**ApoB (SD)**	0.870	0.230	14.311	1	2.386	1.52-3.75	**0.000**	0.185
**Constant**	-0.303	0.198	2.346	1	0.738	-	0.126	
**LDL-C**								
**LDL-C (SD)**	0.645	0.218	8.751	1	1.905	1.24-2.92	**0.001**	0.115
**Constant**	-0.274	0.199	1.897	1	0.761	-	0.168	
					**LDL/ApoB**			
**LDL/ApoB (SD)**	-0.145	0.197	0.546	1	0.865	0.588-1.271	0.460	0.007
**Constant**	-0.269	0.190	1.994	1	0.764	-	0.158	
**Non-HDL-C**								
**Non-HDL-C (SD)**	0.634	0.219	8.390	1	1.884	1.23-2.9	**0.004**	0.106
**Constant**	-0.306	0.192	2.551	1	0.736	-	0.110	
**TC**								
**TC (SD)**	0.598	0.213	9.911	1	1.819	1.2-2.76	**0.003**	0.101
**Constant**							0.196	

Table S4. Bivariate logistic regression: standardized apoB as predictor for left main atherosclerosis and three vessels disease

**Biomarker**	**B**	**SE**	**Wald**	**dF**	**OR**	**95% CI for OR**	**p-value**	**Nagelkerke R^2**
**Left Main disease**								
**ApoB (SD)**	0.889	0.291	9.346	1	2.433	1.38-4.30	**0.002**	0.160
**Constant**	-2.425	0.365	44.195	1	0.088	-	0.002	
**Three vessels disease**								

**ApoB (SD)**	0.421	0.226	3.459	1	1.523	0.98-2.37	0.063	0.045
**Constant**	-1.503	0.0.242	38.616	1	0.223	-	0.000	

Tabel S5. Multivariate logistic regression model of standardized apoB and traditional CV risk factors (age, sex, smoking status, DM, BMI, AF, hypertension and eGFR)

**Biomarker**	**B**	**SE**	**Wald**	**dF**	**OR**	**95% CI for OR**	**p-value**	**Nagelkerke R^2**
**Significant coronary artery disease**								
**ApoB (SD)**	0.919	0.276	11.040	1	2.506	1.458-4.308	**0.000**	0.299
**Three vessels disease**								
**ApoB (SD)**	0.377	0.276	1.862	1	1.458	0.848-2.504	**0.045**	0.221
**Left Main disease**								
**ApoB (SD)**	1.567	0.568	7.612	1	4.792	1.574-14.585	**0.006**	0.416

Table S6. Bivariate logistic regression: comparison between apoB and LDL/ApoB ratio as predictors for coronary atherosclerosis

**Biomarker**	**B**	**SE**	**Wald**	**dF**	**OR**	**95% CI for OR**	**p-value**	**Nagelkerke R^2**
**Coronary atherosclerosis**								
**ApoB (SD)**	1.533	0.356	18.565	1	4.634	2.307-9.309	**0.000**	0.311
**LDL/ApoB (SD)**	-0.593	0.230	6.651	1	0.553	0.352-0.867	**0.010**	0.096

Table S7. Bivariate logistic regression: residual apoB as predictor for coronary atherosclerosis and significant CAD

**Biomarker**	**B**	**SE**	**Wald**	**dF**	**OR**	**95% CI for OR**	**p-value**	**Nagelkerke R^2**
**Coronary atherosclerosis**								
**Residual apoB**	1.653	0.507	10.615	1	5.222	1.932-14.116	**0.001**	0.105
**Constant**	0.405	0.264	2.367	1	1.500	-	**0.124**	
**Significant coronary artery disease**								
**Residual apoB**	0.654	0.384	2.900	1	1.923	0.907-4.080	**0.089**	0.033
**Constant**	-0.547	0.268	4.162	1	0.579	-	**0.041**	

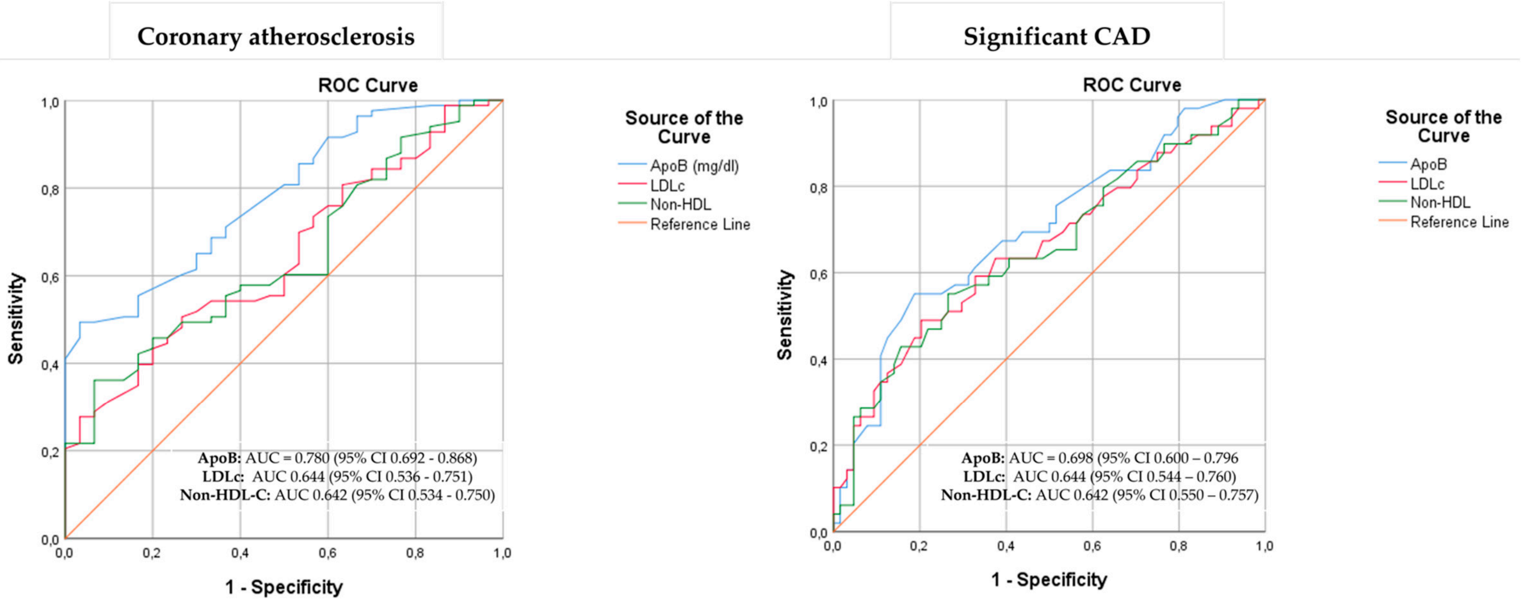

Figure S1. ROC curve showing the association between studied biomarkers and coronary atherosclerosis and significant CAD

Table S8. Echocardiographic findings of the patients included in the study.

Parameter	Overall	N-CAD	NS-CAD	S-CAD	p-Value
			Echocardiographic findings		
LVDD (mm)	50.47 ± 7.25	51.80 ± 9.67	50.00 ± 6.69	50.00 ± 5.88	0.509
IVS (mm)	11.05 ± 1.97	10.90 ± 1.84	10.88 ± 1.87	11.25 ± 2.12	0.619
LVPW (mm)	10.77 ± 1.67	10.67 ± 1.56	10.74 ± 1.52	10.86 ± 1.84	0.869
LVEF (%)	51.49 ± 11.71	49.40 ± 13.19	53.37 ± 11.14	51.44 ± 11.18	0.398
LA indexed Volume (mL/m2)	36.73 ± 17.78	35.04 ± 22.58	35.58 ± 14.35	38.60 ± 16.31	0.626
RA indexed Volume (mL/m2)	26.02 ± 13.84	24.06 ± 16.36	25.57 ± 12.91	27.66 ± 12.61	0.517
LA indexed area (cm^2/m2)	9.01 ± 5.46	7.43 ± 5.55	8.62 ± 5.29	10.32 ± 5.28	0.065
RA indexed area (cm^2/m2)	7.15 ± 4.18	5.77 ± 4.35	7.18 ± 4.18	8.04 ± 3.91	0.067
TRV (m/s)	2.31 ± 0.57	2.29 ± 0.63	2.35 ± 0.57	2.30 ± 0.54	0.903
PAPs (mmHg)	26.86 ± 9.47	28.00 ± 11.74	27.44 ± 9.67	25.85 ± 7.91	0.583
TAPSE (mm)	21.73 ± 3.74	21.17 ± 2.88	22.03 ± 4.76	21.86 ± 3.46	0.637
Aortic atherosclerosis (%)	25.6%	7%	22.2%	44.2%	**0.000**

LVDD: left ventricle diastolic diameter; IVS: interventricular septum; LVPW: left ventricle posterior wall; LVEF: left ventricle ejection fraction; LA: left atrium; RA: right atrium; TRV: tricuspid regurgitation velocity; PAPs:

pulmonary artery systolic pressure; TAPSE: tricuspid annular plane systolic excursion; Ly/neut: lymphocytes to neutrophils ratio;.
